# Multiple gene-to-gene interactions in children with sepsis: a combination of five gene variants predicts outcome of life-threatening sepsis

**DOI:** 10.1186/cc13174

**Published:** 2014-01-02

**Authors:** Petr Jabandziev, Michal Smerek, Jaroslav Michalek, Michal Fedora, Lucie Kosinova, Jaroslav A Hubacek, Jaroslav Michalek

**Affiliations:** 1 Department of Paediatrics, University Hospital Brno, Cernopolni 9, Brno 613 00, Czech Republic; 2 Department of Econometrics, University of Defence, Brno, Czech Republic; 3 Department of Paediatric Anesthesiology and Intensive Care, University Hospital Brno, Brno, Czech Republic; 4 Centre for Experimental Medicine, Institute of Clinical and Experimental Medicine, Prague, Czech Republic; 5 Advanced Cell Immunotherapy Unit, Department of Pharmacology, Faculty of Medicine, Masaryk University, Brno, Czech Republic; 6 Cellthera Ltd, Brno, Czech Republic

## Abstract

**Introduction:**

The aim of the study was to identify the dependency structure of genetic variants that can influence the outcome for paediatric patients with sepsis.

**Methods:**

We evaluated the role of single nucleotide polymorphisms for five genes: bactericidal permeability increasing protein (*BPI*; rs5743507), lipopolysaccharide-binding protein (*LBP*; rs2232618), toll-like receptor 4 (*TLR4*; rs4986790), heat shock protein 70 (*HSP 70*; rs2227956), and interleukin 6 (*IL-6*; rs1800795) in 598 children aged 0 to 19 years that were admitted to a paediatric intensive care unit with fever, systemic inflammatory response syndrome, sepsis, severe sepsis, septic shock, or multiple organ dysfunction syndrome. A control group of 529 healthy individuals was included. Multi-way contingency tables were constructed and statistically evaluated using log-linear models. Typical gene combinations were found for both study groups.

**Results:**

Detailed analyses of the five studied gene profiles revealed significant differences in sepsis survival. Stratification into high-risk, intermediate-risk, and low-risk groups of paediatric patients can predict the severity of sepsis.

**Conclusions:**

Analysis of single nucleotide polymorphisms for five genes can be used as a predictor of sepsis outcome in children.

## Introduction

Sepsis remains one of the most threatening conditions in intensive care units [1,2]. It is defined as the systemic inflammatory response of the human host that is triggered by an invading pathogen (bacterial, viral, fungal, parasitic or combined). Despite outstanding achievements in research and clinical practice, sepsis remains among the major causes of morbidity and mortality worldwide [3,4]. Hitherto, only limited data on this condition have been available from children. A population-based epidemiologic study estimated that in 2003 a total of about 300,000 infectious disease hospitalizations occurred among infants (less than one year of age) in the United States alone and accounted for 42.8% of all infant hospitalizations [5]. Septic states remain one of the most common causes of neonatal morbidity and mortality, especially in the preterm population [6]. The high incidence, associated costs and mortality rate of patients with sepsis has in recent decades led the critical care scientific community to develop specific strategies aimed at improving the outcome of septic states [7,8]. Nevertheless, sepsis mortality has not decreased dramatically during the past decade [4].

Patients admitted to intensive care units with general conditions that seemingly correspond to the severity of infection may nevertheless present fundamentally different survival rates. We hypothesize that at least part of this variability in the sepsis outcome may be due to variation in genes coding components of the innate immune response. Individual differences in disease manifestation influenced by the genetic predisposition have been recognized by the PIRO concept that stratifies patients with sepsis on the basis of their Predisposing conditions, the nature and extent of the Insult (infection or trauma), the nature and magnitude of the host Response, and the degree of concomitant Organ dysfunction [9]. The P, R and O components of the PIRO concept depend largely on the genetic predisposition of the individual patient.

Gene variants (mainly single nucleotide polymorphisms (SNPs)) in genes related to inflammatory and immune system regulations may explain, at least to some extent, the variability of clinical course observed in sepsis and infections. Most previous studies related to SNPs in sepsis were performed on adults [10]. Our group focuses on the paediatric population and previously demonstrated that interleukin 6 (IL-6) and bactericidal permeability increasing protein (BPI) polymorphisms are associated with different outcomes of sepsis [11,12]. We recently have focused on multiparametric analyses of five polymorphisms in five genes that play critical roles in or are associated with inflammatory response, sepsis severity and mortality in order to identify possible predictive mechanisms for sepsis risk stratification. The following genes and their SNPs were investigated: 1) toll-like receptor 4 (*TLR,* OMIM acc. No. 603030) (rs4986790), which is a part of the lipopolysaccharides (LPS) recognition/response unit [13]; 2) lipopolysaccharide-binding protein (*LBP,* OMIM acc. No. 151990) (rs2232618), which is a soluble acute phase protein that binds to LPS of Gram-negative bacteria and facilitates the transfer of bacterial LPS to the specific receptor CD14 [14]; 3) bactericidal permeability increasing protein (*BPI,* OMIM acc. No. 109195) (rs5743507), which displays activity against a wide range of Gram-negative bacteria, reflecting high affinity to lipid A of the LPS regions and potent endotoxin-neutralizing activity [12,15]; and 4) interleukin 6 (*IL-6,* OMIM acc. No. 147620) (rs1800795), which is a key proinflammatory cytokine and plays an important role in the development, pathogenesis and outcome of systemic inflammatory response syndrome, sepsis and septic shock [11]. Plasma levels of IL-6 are elevated in patients with sepsis and high IL-6 concentrations are associated with increased mortality [16,17]. Genetic variation within the regulatory part of the *IL-6* gene may affect the incidence and outcome of sepsis [10,11]. Finally, the role of heat shock protein A1L (*HSP 70*, OMIM acc. No. 140560) (rs2227956), which helps to protect cells from thermal or oxidative stress [18,19], was investigated. While several studies have revealed the importance of genetic polymorphisms in the course and outcome of sepsis [10,20], only a few [21] have demonstrated the influence of combinations of genetic polymorphisms, even though the genetic predisposition to sepsis is polygenic and with many variants in multiple gene loci playing different roles. Only limited data are available from the paediatric sepsis population regarding genetic polymorphism studies and their role in sepsis severity prediction. To our knowledge, no multiple gene SNPs analysis has been performed to demonstrate predictability in paediatric patients with sepsis. This study presents data based on multiparametric analyses of five SNPs of immune-related and inflammation-related genes that are involved in the immune response in sepsis.

## Materials and methods

A total of 598 paediatric patients aged 0 to 19 years (325 males, 273 females) were enrolled if they met the following inclusion criteria: 1) admission to the Paediatric Intensive Care Unit (PICU) at University Hospital Brno for at least 24 h; 2) fever (defined as body temperature above 39°C or above 38.5°C measured consecutively at two occasions at least 6 h apart), systemic inflammatory response syndrome, sepsis, severe sepsis, septic shock or multiple organ dysfunction syndrome (MODS) based on generally accepted consensus criteria published and modified for paediatric patients by the American College of Chest Physicians and the Society of Critical Care Medicine [22,23]; 3) signed informed consent by their parents or legal guardians; and 4) successful genotyping of all studied gene variants.

Patients were enrolled from September 2003 to December 2009, their clinical status was monitored on a daily basis, and the outcome of the stay at the PICU was carefully evaluated. Patients hereinafter referred to as non-survivors died as a direct consequence of the septic event.

As a control group, 529 healthy individuals (269 male and 260 female) aged 26 to 67 years were analysed after signing a written informed consent. This group represents a random sample of the population of two districts of the Czech Republic selected according to the World Health Organization protocol (Multinational monitoring of trends and determinants in cardiovascular diseases (the MONICA Project)). This study hereinafter refers to patients generally as the patient group (PG), patients with severe sepsis, septic shock, or MODS as the patient group with severe condition (PGS), and healthy controls as the control group (CG).

This study was approved by the Institutional Review Board of the University Hospital Brno in accordance with the 1964 Declaration of Helsinki.

### Genetic analysis

DNA was isolated according to the standard protocol from EDTA blood as previously described [24]. DNA variants of the genes studied (that is, *BPI*, *LBP*, *TLR*, *HSP 70* and *IL-6*) were analysed using PCR and restriction analyses. For more details about the oligonucleotide sequences, restriction enzyme used and detailed PCR conditions, see the authors’ previous work [11,12] or contact the corresponding author.

### Statistical methods

The statistical theory of log-linear models and logit analysis for the evaluation of multi-way contingency tables was used [25], including the theory of graphical models [26]. The adequate log-linear graphical model was chosen by stepwise procedure [27] using STATISTICA software manufacturer: (StatSoft Inc., Tulsa, OK, USA) and likelihood ratio statistics G^2^. The U statistics based on arcsine transformation [28] and Fisher’s exact test with mid-*P*-value [25] were used to compare the equality of two independent binomial populations (frequencies) for small sample sizes. The theory of generalized linear models [25] was used to classify the combinations of risk SNP variants into groups according to the level of risk and to calculate probabilities for the risk groups. The software STATISTICA (version 10.0.1011.0) and MATLAB software manufacturer: (MathWorks Inc., Natick, MA, USA) (version 7.11.0.584) were used for computing.

### Statistical analysis

Descriptive statistics were used for basic characterization of both PG and CG. Individual genotypes were distinguished and labelled as follows: major/common homozygote (aa), heterozygote (ab) and minor allele homozygote (bb). Some genotype frequencies in group (bb) were very small, including only three or fewer subjects. For this reason, it was necessary to re-code the gene variants to meet the requirements of the statistical tests and enable reliable evaluation of information from the data sets. Thus, genotypes of all studied genes were coded and labelled according to the following key: common, major homozygotes (aa) were labelled (A) and those remaining (that is, heterozygotes (ab) and minor homozygotes (bb)) were collapsed and labelled (B). Absolute and relative frequencies were determined for the occurrence of variant A of the respective gene in PG, PGS and CG.

Further analyses were based on comparisons of relative frequencies between PG and CG or between PGS and CG. These comparisons were performed not only for individual SNPs of each gene, but also for the combinations of two, three, four and five genes. Initially, two five-way contingency tables (for each PG and CG) were created. Each table was formatted as 2 × 2 × 2 × 2 × 2 for the five SNPs studied. These contingency tables were then analysed using log-linear models for five dimensions, the optimal graphical model for both groups was found, and comparisons of groups were made using Fisher’s exact test and mid-*P*-value as well as by the test using U statistics for comparing two independent binomial frequencies. Two different statistics were used to compare the adequacy of the chosen tests in cases of small frequencies. When both tests yielded the same results, only results based on Fisher’s exact test are reported. An identical approach was then applied for comparisons between PGS and CG.

Based on results of previous comparisons, the theory of generalized linear models was used and the SNP combinations were classified according to the value of the probability that the given SNP combination would appear in PG or CG. The SNP combination risk groups could be determined using this method. If the results of all methods are consistent, typical SNP variants associated with high risk and low risk of sepsis outcome could be identified and described.

## Results

### Patient clinical characteristics

Most of the patients were admitted to the PICU due to infection (181 patients with respiratory tract infection (30.3%), 60 with urogenital infection (10%), 73 with central nervous system infection (12.2%), 56 with abdominal infection (9.4%) and 49 with other infections (8.2%)). The remaining 179 (29.9%) were admitted due to trauma or some other surgical condition. Overall survival was 575 (96.2%) out of 598 patients enrolled. All patients experiencing only a febrile episode (131 patients) or systemic inflammatory response syndrome (314 patients) survived. As expected, the mortality rate was low in the sepsis subgroup (2.6%, that is, 1 out of 39 patients), higher in the severe sepsis subgroup (5.5%, that is, 4 out of 73 patients), and highest in the septic shock and MODS subgroup (43.9%, that is, 18 out of 41 patients). Detailed characteristics of non-survivors are summarized in Table 1. The presence of an infection either upon admission or that developed during the stay at the PICU was confirmed in 297 (49.7%) of 598 paediatric patients. The cause of infection was Gram-positive bacteria in 123 cases (41.4%), Gram-negative bacteria in 112 cases (37.7%), viruses in 37 cases (12.5%) and fungi or other infectious agents in 25 cases (8.4%).

**Table 1:** Clinical and SNP characteristics of non-survivors

**Original diagnosis**	**Cause of death**	**Causative pathogen**	***BPI***	***LBP***	***TLR***	***HSP 70***	***IL-6***	**Risk variants of five genes**
Crohn’s disease	Septic shock	CMV, Candida	A	A	A	B	A	H
Pneumonia	Septic shock	Not identified	A	A	A	B	A	H
Peritonitis	Septic shock	Not identified	A	A	A	A	B	H
Pneumonia	Severe sepsis	G + bacteria	A	B	A	A	B	I
Pneumonia	Septic shock	Not identified	A	A	A	A	B	H
Pneumonia	Septic shock	Not identified	A	B	A	A	A	H
Pneumonia	Septic shock	G- bacteria	A	A	A	A	B	H
Pneumonia	Severe sepsis	G + bacteria	A	B	A	B	B	I
Multiple injury	Septic shock	G + bacteria	A	A	A	A	B	H
Pneumonia	Septic shock	Not identified	A	A	A	B	A	H
Pneumonia	Severe sepsis	G- bacteria	A	A	A	B	A	H
Pneumonia	Septic shock	Not identified	A	A	A	A	A	I
Ileus	Septic shock	G + bacteria	A	A	A	A	B	H
Multiple injury	Sepsis	Not identified	A	A	A	A	B	H
Pneumonia	Severe sepsis	Not identified	B	A	A	B	B	I
Cranial injury	Septic shock	G + bacteria	A	A	A	A	B	H
Pneumonia	Septic shock	Aspergillus	A	B	A	B	B	I
Meningitis	Septic shock	Not identified	B	A	B	A	A	I
Pulm. embolism	Septic shock	Actinomyces	A	A	A	A	B	H
Pneumonia	Septic shock	G + bacteria	A	A	B	A	A	I
Pneumonia	Septic shock	Candida	A	B	B	A	B	I
Ileus	Septic shock	G- bacteria	A	B	A	A	B	I
Gastroenteritis	MODS	G- bacteria	A	A	A	A	B	H

A, common (wild-type) homozygotes (aa); B, heterozygotes (ab) and minor homozygotes (bb); H, high-risk sepsis combination of five single nucleotide polymorphism (SNP) variants; I, intermediate risk sepsis combination of five SNP variants.

### Single nucleotide polymorphisms

**Figure 1:**
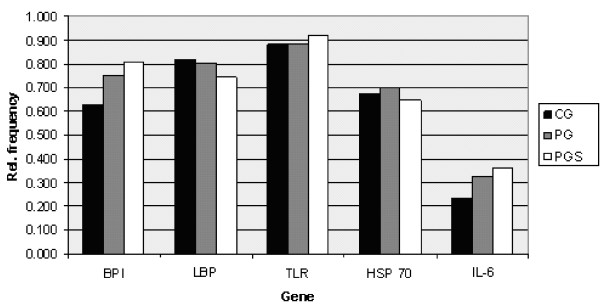
**Relative frequencies of wild-type homozygote variants.** Relative frequencies of the occurrence of major homozygotes for the given genes in the control group (CG), group of patients (PG) and group of patients with highly serious sepsis - severe sepsis, septic shock and multiple organ dysfunction syndrome (MODS) (patient group with severe condition, PGS).

The complete genotyping was successful in 598 patients and in 529 control individuals. No age or gender differences have been demonstrated in the distribution of gene variants in either PG or CG. The distributions of individual genotypes of both polymorphisms are in Hardy-Weinberg equilibrium in both groups. The genotype relative frequencies in PG and CG are shown in Figure 1 and Table 2.

**Table 2:** Wild type homozygote frequencies

**Gene**	**CG (n = 529)**		**PG (n = 598)**		**PGS (n = 114)**	
	**abs.**	**rel.**	**abs.**	**rel.**	**abs.**	**rel.**
*BPI*	333	0.629	450	0.753	92	0.807
*LBP*	432	0.817	480	0.803	85	0.746
*TLR*	466	0.881	531	0.888	105	0.921
*HSP 70*	355	0.671	420	0.702	74	0.649
*IL-6*	124	0.234	194	0.324	41	0.360

Absolute (abs.) and relative (rel.) frequencies of the occurrence of major homozygotes for the given genes in the control group (CG), group of patients (PG), and group of patients with highly serious sepsis - severe sepsis, septic shock and multiple organ dysfunction syndrome (MODS) (patient group with severe condition, PGS).

**Figure 2:**
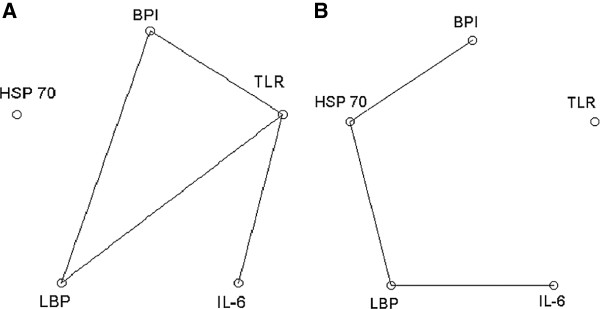
**Optimal graphical models of genetic interconnections.** Optimal association graphs describing relations among five single nucleotide polymorphisms (SNPs) in patients and healthy controls. **A)** Model mapping the most typical associations between genes studied in group of patients (PG). **B)** Model mapping the most typical associations between genes studied in control group (CG).

To describe interactions among the five genes studied, the adequate statistical association structures of SNPs in both PG and CG were determined using optimal association graphs. Associations are demonstrated in Figure 2. Both graphs were constructed to reveal the most typical statistical associations among the SNPs studied. The differences between PG and CG are demonstrated at Figure 1, Figure 2 and Table 2. For PG, the association among *BPI*, *TLR* and *LBP* is typical and there is a three-factor interaction. This means that each pair of these three variables may be conditionally dependent and an odds ratio for any pair of these three variables may vary across levels of the third variable. In addition, *IL-6* is conditionally independent from *BPI* and *LBP* provided that the presence of the *TLR* SNP is fixed, whereas the occurrence of the *HSP 70* SNP is independent of the preceding four genes. In CG, associations are described between *BPI* and *HSP 70*, *HSP 70* and *LBP*, and *LBP* and *IL-6*. The *TLR* SNP is independent from the other four SNPs. The graphs in Figure 2 were used in further searching for high-risk and low-risk variants of SNP combinations.

In view of Figure 2, detailed comparison was made between the frequencies of the following three SNP combinations: 1) *BPI*, *LBP* and *TLR*; 2) *BPI*, TLR and *IL-6*; and 3) *LBP*, *TLR* and *IL-6*. Binomial testing based on U statistics and Fisher’s exact test was used. The results comparing PG and CG as well as PGS and CG demonstrated significant differences, and the findings of the two statistical tests were in good agreement. Therefore, only the results of Fisher’s exact test are reported in Table 2. Table 2 also demonstrates risk prediction in unrelated *BPI* and *HSP 70* SNPs as well as non-associated combination of *BPI* and *HSP 70* and combination of all five examined SNPs. Based on these results, we can clearly identify high, intermediate and low sepsis risk populations in paediatric patients.

Detailed statistical analysis of a unique *BPI*, *LBP* and *TLR* gene triplet detected significant differences in genetic structure between the studied groups. The high-risk combination associated with sepsis development was BPI A + LBP A + TLR A. The proportion of combined major homozygotes was significantly higher in PG compared to CG (*P* = 0.005). The comparison between the PGS and the CG revealed BPI A + LBP B + TLR A as a high-risk combination (*P* = 0.034). On the contrary, a low-risk sepsis development combination was BPI B + LBP A + TLR A for both PG and PGS in comparison with CG (*P* <0.001; *P* = 0.003, respectively). Similar to the previous triplet, the combination of major homozygosity for *BPI*, *TLR* and *IL-6* SNPs was associated with high risk of sepsis development (*P* <0.001). Furthermore, this association was even more expressed in PGS (*P* = 0.003). In contrast, the combination of BPI B + TLR A + IL-6 B represents low risk for PG and PGS (*P* <0.001; *P* = 0.001, respectively).

Analyses of the specific triplet *LBP*, *TLR* and *IL-6* showed LBP A + TLR B + IL-6 A and LBP B + TLR A + IL-6 A combinations (*P* = 0.006; *P* = 0.012, respectively) to be at high risk for sepsis development, but occurrence of the proposed variants was low (4.2 and 4.8% of patients). A low-risk variant for PGS was the LBP A + TLR A + IL-6 B combination (*P* = 0.027), which was the most common variant in this group (41.2% of patients).

A non-associated combination of wild-type homozygote variants of *BPI* and *HSP 70* genes together represented high risk for sepsis development (*P* < 0.001), and this was statistically significant for PGS (*P* = 0.004). The combination BPI B + HSP 70 A for the sepsis group (*P* <0.001) and for PGS (*P* <0.001) represented low risk.

This highly complex analysis of the five studied genes’ distribution showed significant differences between patients and control groups. A high-risk combination for sepsis development and typical for the patients group was the quintet of wild-type homozygotes (*P* = 0.005), but the most common specific combination in the three studied groups was BPI A + LBP A + TLR A + HSP 70 A + IL-6 B. This combination also represented high risk for sepsis development (*P* = 0.016). A low-risk variant was BPI B + LBP A + TLR A + HSP 70 A + IL-6 B both in patients (*P* = 0.006) and PGS (*P* = 0.001). Frequencies of other low-risk variants BPI B + LBP A + TLR B + HSP 70 A + IL-6 B and BPI B + LBP B + TLR A + HSP 70 A + IL-6 B differ significantly between the groups (for details, see Table 3).

**Table 3:** Evaluation of sepsis risk based on five SNPs in paediatric patients

			**Control group (n = 529)**		**Patient group (n = 598)**				**Patient group with severe condition (n = 114)**			
	**Genes**	SNP* variant	**No.**	**(%)**	**No.**	**(%)**	**Fisher Mid-*P***	**Risk**	**No.**	**(%)**	**Fisher Mid-*P***	**Risk**
Associated SNP combinations	*BPI + LBP + TLR*	AAA	237	43.5	319	53.3	0.005	H	62	54.4	0.071	I
		AAB	31	5.9	34	5.7	0.950	I	4	3.5	0.236	I
		ABA	58	11.0	87	14.5	0.068	I	23	20.2	0.034	H
		ABB	7	1.3	10	1.7	0.721	I	3	2.6	0.308	I
		BAA	144	37.8	110	18.4	<0.001	L	17	14.9	0.003	L
		BAB	20	3.8	17	2.8	0.361	I	2	1.8	0.224	I
		BBA	27	5.1	15	2.5	0.022	L	3	2.6	0.198	I
		BBB	5	0.9	6	1.0	0.882	I	0	0	0.188	I
	*BPI + TLR + IL-6*	AAA	69	13.0	127	21.2	<0.001	H	31	27.2	0.003	H
		AAB	226	42.7	279	46.7	0.177	I	54	47.4	0.378	I
		ABA	7	1.3	21	3.5	0.016	H	1	0.9	0.818	I
		ABB	31	5.9	23	3.8	0.109	I	6	5.3	0.745	I
		BAA	44	8.3	36	6.0	0.148	I	7	6.1	0.398	I
		BAB	127	24.0	89	14.9	<0.001	L	13	11.4	0.001	L
		BBA	4	0.8	10	1.7	0.146	I	2	1.8	0.489	I
		BBB	21	4.0	13	2.8	0.100	I	0	0	0.008	L
	*LBP + TLR + IL-6*	AAA	102	19.3	134	22.4	0.200	I	32	28.1	0.050	I
		AAB	279	52.7	295	49.3	0.245	I	47	41.2	0.027	L
		ABA	8	1.5	25	4.2	0.006	H	3	2.6	0.602	I
		ABB	43	8.1	26	4.3	0.011	L	3	2.6	0.015	L
		BAA	11	2.1	29	4.8	0.012	H	6	5.3	0.206	I
		BAB	74	14.0	73	12.2	0.401	I	20	17.5	0.345	I
		BBA	3	0.6	6	1.0	0.416	I	0	0	0.278	I
		BBB	9	1.7	10	1.7	0.909	I	3	2.6	0.596	I
Non-associated SNP combination	*BPI + HSP 70*	AA	212	40.1	323	54.0	<0.001	H	63	55.3	0.004	H
		AB	121	22.9	127	21.2	0.494	I	29	25.4	0.585	I
		BA	143	27.0	97	16.2	<0.001	L	11	9.6	<0.001	L
		BB	53	10.0	51	8.5	0.382	I	11	9.6	0.932	I
All SNPs in combination	*BPI + LBP + TLR + HSP 70 + IL-6*	AAAAA	36	6.8	70	11.7	0.005	H	13	11.4	0.157	I
		AAAAB	111	21.0	162	27.1	0.016	H	29	25.4	0.354	I
		AABBB	14	3.6	3	0.5	0.004	L	1	0.9	0.172	I
		ABAAA	6	1.1	22	3.7	0.005	H	5	4.4	0.133	I
		BAAAB	70	13.2	48	8.0	0.006	L	4	3.5	0.001	L
		BABAB	15	2.8	7	1.2	0.041	L	0	0	0.026	L
		BBAAB	20	3.8	8	1.3	0.009	L	2	1.8	0.224	I
		others	257	48.6	278	46.5	0.492	I	60	52.6	0.440	I

Single nucleotide polymorphisms’ (SNPs’) frequencies and their sepsis risk evaluation for three sets of associated genes, non-associated genes and five gene SNPs.

SNP variants indicate the wild-type (most frequent) homozygote with label “A”, while heterozygote or minor homozygotes are shown as “B”. SNP variants are stated in the same order as genes.

These data are in agreement with results of the other statistical method, the generalized linear model (optimal logit model). These models calculated the probabilities that individuals with defined combinations of SNPs belong to CG or PG. The logit model then enables setting 95% confidence intervals for these probabilities. The results of these analyses confirmed that all combinations for high-risk and low-risk cases shown in Table 3 are adequate (see the Additional file 1 for further details).

Finally, in the group of patients with severe condition, a comparison was made between survivors and non-survivors using the Fisher’s exact test and mid-*P*-values. Due to limited numbers in each subgroup, the power of the tests used is low and the data demonstrate only trends or statistical significance at the 10% level (Table 4). The one-side and two-side alternatives were considered.

**Table 4:** Risk of death in patients with severe condition

**Genes**	**SNP variant**	**Survivors (n = 91)**		**Non-survivors (n = 23)**		***P *** **(one)**	***P *** **(both)**	**Risk of death SNP variant**
		**No.**	**(%)**	**No.**	**(%)**			
*BPI*	A	71	78.0	21	91.3	0.077	0.088	Risk
*BPI*	B	20	22.0	2	8.7	0.077	0.088	Non-risk
*BPI; TLR*	BA	19	20.9	1	4.3	0.029	0.029	Non-risk
*TLR; IL-6*	BA	1	1.1	2	8.7	0.055	0.56	Risk
*BPI; LBP; TLR*	BAA	16	17.6	1	4.3	0.056	0.062	Non-risk
*BPI; TLR; IL-6*	BAA	7	7.7	0	0	0.098	0.098	Non-risk
*LBP; TLR; IL-6*	ABA	1	1.1	2	8.7	0.055	0.56	Risk
*BPI; LBP; TLR; HSP 70; IL-6*	AAAAB	20	22.0	9	39.1	0.055	0.155	Risk
	AAABB	8	8.8	0	0	0.077	0.077	Non-risk

All single nucleotide polymorphism (SNP) combinations revealing at least 10% level of statistical significance between survivors and non-survivors of severe sepsis, septic shock or multiple organ dysfunction syndromes (MODS) (based on two-sided Fisher’s exact test) are shown. Other SNP combinations did not demonstrate statistically significant differences and are not presented.

The distribution showed no differences in any patient group when compared to the control group. However, specific combinations indicated a tendency to be overrepresented in the non-survivors group, such as *BPI* major homozygotes (*P* = 0.077), combination of TLR B and IL-6 A (*P* = 0.055), combination of LBP A + TLR B + IL-6 A (*P* = 0.055) and BPI A + LBP A + TLR A + HSP 70 A + IL-6 B (*P* = 0.055).

## Discussion

The immune response in sepsis is an extremely multifaceted cascade of events involving inflammatory and anti-inflammatory processes, humoral and cellular reactions, and circulatory abnormalities [29]. Several risk factors for sepsis development have been identified [30], but the cause of basic differences in susceptibility between individuals and populations remains unclear.

Host genetic variability in the regulatory and coding regions of genes for components of the innate immune system, inflammatory cytokines and coagulation cascade may influence the susceptibility to and/or outcome from sepsis. SNPs can result in absolute deficiency of a protein, an altered protein, a change in the level of normal protein expression, or no discernible change in protein function or expression, and they are thought to explain at least in part the interindividual differences in susceptibility [30].

In recent years, many investigators have observed potential associations between immune-related gene polymorphisms and the development, course and outcome of septic episodes - and often with apparently conflicting results [10,20]. Differences in study design, ethnicity, as well as gene-to-gene and gene-to-environmental interactions could be limiting factors. The outcome of a septic condition is influenced by multiple host and pathogen factors, including the patient’s age, gender and race, as well as the presence of comorbid conditions, the patient’s underlying immune status, and the specific pathogen involved [31]. It is clear that only a part of the genetic contribution to sepsis development may be explained by the identified gene polymorphisms.

Differences in environmental exposures and genetic heterogeneity between ethnic groups may have complicated the search for genetic and gene-environmental determinants. The contributions of gene-gene interaction to the risk of diseases have been documented (for example, in the case of breast cancer) [32,33]. Data from populations with sepsis are poor and, moreover, developmental differences that affect the haemodynamic, inflammatory, coagulation and immune responses make it difficult to extrapolate data from adult studies to paediatric populations [31].

This study evaluates combined genetic polymorphisms for their possible association with susceptibility to septic conditions and outcome of all septic episodes. To the best of our knowledge, we have evaluated for the first time in a Central European population of critically ill children the influence of genetic polymorphism interactions of five genes related to immune response. The results demonstrate that the specific combinations of genetic polymorphisms seem to be associated with sepsis development. We believe that we can conclude this, despite the fact that we have used healthy adults as controls. There is no evidence that allelic frequencies in some genes are significantly different in children and in adults.

The study points out the importance of interactions among the *BPI*, *LBP*, *TLR*, *HSP 70* and *IL-6* polymorphisms and also highlights the relevance of the combination of gene polymorphisms to sepsis outcome. Specific combinations of common homozygosity for *BPI*, *LBP*, *TLR*, *HSP 70* and *IL-6* variants were typical within the septic group and were associated with a high risk of sepsis development. Generally, in the group of children with sepsis studied, individuals carrying wild-type alleles of the proposed genes in various combinations had increased risk for sepsis development compared to those with the minor alleles. Similar to our findings, a study by Flores *et al*. [34] had concluded that a common SNP risk haplotype of *LBP* was strongly associated with susceptibility to severe sepsis and homozygous carriers of the risk haplotype had increased risk for severe sepsis.

Benermo *et al*. revealed that the G allele of 174 G>C SNP in the promoter region of the *IL-6* gene is functional *in vivo* with increased inflammatory response [35]. This result could be consistent with the fact that early increased and uncontrolled release of cytokines (known as a cytokine storm) and proinflammatory mediators are peculiar for sepsis development and associated with increased mortality [16,17].

Our data indicate that specific combinations of gene polymorphisms - most frequently wild-type homozygote variants - were significantly associated with sepsis development in a large cohort of paediatric patients. In addition, we revealed significant associations between genetic structure in patients with severe septic conditions (severe sepsis, septic shock and MODS). Moreover, low-risk variants, typical for the control group and representing low risk for sepsis development were also described. Our hypothesis arising from our previous genetic observations states that mutated variants of gene polymorphisms seem to be protective against sepsis development. The exact protective mechanism is unknown due to an incomplete understanding of the complex pathophysiologic nature of sepsis development. Compared to non-carriers, however, volunteers with the mutated *TLR 299* allele have been shown to have lower concentrations of some of the inflammatory cytokines, acute-phase reactants and other mediators of inflammation relatively late after the onset of experimental endotoxemia [36].

## Conclusions

Future genome-wide expression profiling studies will allow researchers to reveal specific interactions between polymorphisms in genes involved in innate immunity and to stratify patients according to their risk for certain outcomes. The first reported genome-wide comparison of expression patterns among healthy children and children with septic shock revealed more than 2,000 genes that were differentially expressed or repressed in patients experiencing septic shock relative to healthy controls [37]. It is only a question of time before a reasonable number of risky combinations for sepsis development and for poor outcomes can be determined [30]. High-risk patients could benefit from being so identified through early introduction of specific preventive or therapeutic interventions.

These data demonstrate that such an approach can clearly identify SNP variants that are associated with favourable and unfavourable sepsis outcomes.

## Key messages

• Five single nucleotide polymorphisms of genes involved in inflammation can stratify paediatric patients for risk of sepsis survival.

• Stratification into high-, intermediate- and low-risk groups of paediatric patients can predict the severity of sepsis.

## Abbreviations

BPI: Bactericidal permeability increasing protein; CG: Control group; HSP 70: Heat shock protein A1L; IL-6: Interleukin 6; LBP: Lipopolysaccharide-binding protein; LPS: Lipopolysaccharide; MODS: Multiple organ dysfunction syndrome; PG: Patient group; PGS: Patient group with severe condition - severe sepsis septic shock or multiple organ dysfunction syndrome; PICU: Paediatric intensive care unit; SNP: Single nucleotide polymorphisms; TLR: Toll-like receptor 4.

## Competing interests

The authors declare that they have no competing interests.

## Authors’ contributions

PJ collected data, managed the study database and composed the manuscript. MS and JM Sr performed the statistical analyses and helped with designing the study. MF interpreted the clinical characteristics of patients. LK collected data and interpreted the clinical characteristics of patients. JAH carried out the molecular genetic studies, analyzed the data, interpreted the results and proofread the manuscript. JM Jr designed and supervised the study and wrote the manuscript. All authors read and approved the final manuscript.

## Supplementary Material

Additional file 1: Table S1 Results based on the optimal graph model. Click here for file
