# Supplementary material for: Multiple gene-to-gene interactions in children with sepsis: a combination of five gene variants predicts outcome of life-threatening sepsis
Source: Crit Care. 2014 Jan 2;18(1):R1. doi: 10.1186/cc13174 (PMC4056441; doi:10.1186/cc13174)
Supplement: Additional file 1: Table S1 — Results based on the optimal graph model. [file cc13174-S1.doc]

**Supplementary table.** Results based on the optimal graph model

| Combination of 5 studied genes | Generalized linear model | | | Absolute frequency | | | Risk |
| --- | --- | --- | --- | --- | --- | --- | --- |
|  | Probability | Lower bound | Upper bound | CG | PG | Sum |  |
| AABAA | 0.213 | 0.116 | 0.359 | 5 | 13 | 18 | High risk |
| AAAAA | 0.339 | 0.276 | 0.408 | 36 | 70 | 106 | High risk |
| ABAAA | 0.292 | 0.177 | 0.442 | 6 | 22 | 28 | High risk |
| AAAAB | 0.403 | 0.353 | 0.455 | 111 | 162 | 273 | High risk |
| AABBB | 0.622 | 0.500 | 0.730 | 14 | 3 | 17 | Low risk |
| BAAAB | 0.604 | 0.534 | 0.670 | 70 | 48 | 118 | Low risk |
| BABAB | 0.679 | 0.565 | 0.775 | 15 | 7 | 22 | Low risk |
| BBAAB | 0.685 | 0.584 | 0.772 | 20 | 8 | 28 | Low risk |
| Sum |  |  |  | 529 | 598 | 1127 |  |

Data were analyzed using generalized linear models. The method is asymptotic and the results are consistent with Fisher's exact test. The Supplementary table shows the results based on the optimal graph model [14,24,25,35]. When the 95% confidence interval for the inclusion of the patient in the CG had the upper limit of less than 0.5, the appropriate gene combinations were marked as High risk. When the 95% confidence interval for the inclusion of patients in the CG had a lower limit greater than 0.5, the appropriate gene combinations were marked as Low risk. Other gene combinations were classified as Intermediate risk. The results are in accordance with Table 3.
